# Molecular diagnosis of patients with epilepsy and developmental delay using a customized panel of epilepsy genes

**DOI:** 10.1371/journal.pone.0188978

**Published:** 2017-11-30

**Authors:** Laura Ortega-Moreno, Beatriz G. Giráldez, Victor Soto-Insuga, Rebeca Losada-Del Pozo, María Rodrigo-Moreno, Cristina Alarcón-Morcillo, Gema Sánchez-Martín, Esther Díaz-Gómez, Rosa Guerrero-López, José M. Serratosa, Grupo Español de Genética de las Epilepsias de la Infancia (GEGEI)

**Affiliations:** 1 Neurology Lab and Epilepsy Unit, Department of Neurology, IIS- Fundación Jiménez Díaz, UAM, Madrid, Spain; 2 Centro de Investigación Biomédica en Red de Enfermedades Raras (CIBERER), Madrid, Spain; 3 Department of Pediatrics, Hospital Universitario Fundación Jiménez Díaz, UAM, Madrid, Spain; University of Catanzaro, ITALY

## Abstract

Pediatric epilepsies are a group of disorders with a broad phenotypic spectrum that are associated with great genetic heterogeneity, thus making sequential single-gene testing an impractical basis for diagnostic strategy. The advent of next-generation sequencing has increased the success rate of epilepsy diagnosis, and targeted resequencing using genetic panels is the a most cost-effective choice. We report the results found in a group of 87 patients with epilepsy and developmental delay using targeted next generation sequencing (custom-designed Haloplex panel). Using this gene panel, we were able to identify disease-causing variants in 17 out of 87 (19.5%) analyzed patients, all found in known epilepsy-associated genes (*KCNQ2*, *CDKL5*, *STXBP1*, *SCN1A*, *PCDH19*, *POLG*, *SLC2A1*, A*RX*, *ALG13*, *CHD2*, *SYNGAP1*, and *GRIN1)*. Twelve of 18 variants arose *de novo* and 6 were novel. The highest yield was found in patients with onset in the first years of life, especially in patients classified as having early-onset epileptic encephalopathy. Knowledge of the underlying genetic cause provides essential information on prognosis and could be used to avoid unnecessary studies, which may result in a greater diagnostic cost-effectiveness.

## Introduction

Epilepsy is a common neurologic disorder in childhood, with a prevalence of 300–600 per 100000. About 30% of children with epilepsy present behavioral or cognitive impairment [1]. Among the most severe forms of childhood epilepsy are the so-called epileptic encephalopathies (EEs), which include a number of heterogeneous early-onset clinical disorders characterised by refractory seizures, developmental delay, or regression associated with ongoing epileptic activity, and poor prognosis in the majority of the patients [2, 3]. Dravet, Ohtahara, and West syndromes are some of the most common EEs [2]; however, many neonates and infants with EEs do not fit into any of the proposed epileptic syndromes [4].

With the advancement of technologies for genetic diagnosis, genetic defects have been increasingly recognised as causes of different types of pedriatric epilepsies, and also seem to account for a significant number of EEs [5, 6]. Genes involved in ion channelopathies, neuronal transmission, brain development, or synaptic functions have been reported to be associated with EE [7]. To date, more than 500 genes have been linked to epilepsy, and several genes—including *STXBP1*, *ARX*, *SLC25A22*, *KCNQ2*, *CDKL5*, *SCN1A*, and *PCDH19*—have been found to be associated with EEs [8–11]. The genetic and phenotypic heterogeneity in pediatric epilepsies [12–20] coupled with the fact that very few cases are explained by mutations in the same gene [21] make sequential single-gene testing impractical. Genetic testing panels open new possibilities for the diagnosis of this type of epilepsies, especially those for which diagnosis is otherwise unclear [8, 22–27].

The aim of this study was to perform a comprehensive genetic analysis using next-generation sequencing (NGS) technology to analyze more than 80 genes previously associated with epilepsy in 87 patients with epilepsy and developmental delay.

## Materials and methods

### Patients

We selected 87 patients with epilepsy and developmental delay of unexplained origin referred to our laboratory for genetic study. Most patients had been previously studied in order to rule out a structural or metabolic etiology. Patients’ medical histories and results from laboratory testing were obtained by face-to-face interview and by consulting medical records. When available, information regarding the patient’s clinical, imaging, and neurophysiology data were reviewed by 2 epileptologists with experience in clinical epilepsy genetics, and phenotypes were classified into known electroclinical syndromes. When there was insufficient information, or the phenotype did not correspond to any recognizable syndrome, patients were included in the unclassified group. Before performing the genetic panel, 41 (47.1%) patients had been studied for mutations in selected epilepsy genes with conventional techniques (see Table 1).

**Table 1:** Mutations identified by a multigenic panel of epilepsy.

ID	Sex	Phenotype	Age at seizure onset	Previous genetic analysis	Gene / Transcript	Variant	dbSNP147 / MAF	Inheritance	PolyPhen2 / SIFT (score)	GERP (score)	ExAC (Allele frequency)
1	F	EOEE	2 days	PNPO STXBP1	KCNQ2 / NM_004518.5	c.602G>A / p.Arg201His	Reported by Carvill et al., 2013	IVF	0.979 / 0	3.88	Not present
2	F	EOEE	NA	None				*De novo*			
3	M	EOEE	17 hours	None		c.601C>T / p.Arg201Cys	rs796052623 / NA	IVF	0.979 / 0	2.84	Not present
4	M	Unclassified EE	24 hours	None		c.803T>C / p.Leu268Pro	rs864321708 / NA	*De novo*	0.053 / 0.03	3.38	Not present
5	F	EOEE	20 days	KCNQ2 PRRT2 SCN2A	STXBP1 / NM_001032221.3	c.1216C>T / p.Arg406Cys	rs796053367 / NA	*De novo*	0.923 / 0	5.61	Not present
6	F	NLES	5 months	ARX CDKL5 SCN1A	ALG13 / NM_001039210.4	c.320A>G / p.Asn107Ser	rs398122394 / NA	*De novo*	0.869 / 0	2.13	Not present
7	M	Unclassified EE	1 months	KCNQ2	CDKL5 / NM_001037343.1	c.52_53insT / p.Val19CysfsTer3	Not reported	*De novo*	NA	5.56	Not present
8	F	Unclassified EE	6 months	None		c.377G>A / p.Cys126Tyr	Reported by Fehr et al., 2015	*De novo*	0.998 / 0	5.98	Not present
9	F	Unclassified EE	1 months	None		c.533G>A / p.Arg178Gln	rs267606715 / NA	*De novo*	1 / 0	5.60	Not present
10	F	SMEI	6 months	None	PCDH19 / NM_001105243.1	c.698A>G / p.Asp233Gly	Not reported	Paternally inherited	0.999 / 0	6.08	Not present
11	F	Unclassified EE	4 months	PCDH19	SCN1A / NM_001165963.1	c.602+1G>A	rs794726827 / NA	*De novo*	NA	5.24	Not present
12	M	Unclassified EE	6 months	SLC2A1 SYNGAP1	CHD2 / NM_001042572.2	c.2317G>A / p.Glu773Lys	Not reported	Parents not available	0.019 / 0.28	5.18	Not present
13	F	Unclassified EE	2 months	SLC2A1	SLC2A1 / NM_006516.2	c.115-2A>G	Not reported	*De novo*	NA	5.24	Not present
14	M	Unclassified EE	18 months	SCN1A SLC2A1	SYNGAP1 / NM_001130066.1	c.333_334insG / p.Lys114GlufsTer38	Not reported	*De novo*	NA	-2.27	Not present
15	M	EOEE	1 months	None	ARX / NM_139058.2	c.196G>A / p.Gly66Ser	rs1057518564 / NA	*De novo*	0.788 / 0.21	4.90	Not present
16	M	Unclassified EE	3 years	SRPX2	POLG / NM_001126131.1	c.156_158dupGCA / p.Gln52dup	rs41550117 / NA	Maternally inherited	NA	0.00	0.01921
						c.2492A>G / p.Tyr831Cys	rs41549716 / 0.02	Parents not available	0.948 / 0.07	1.47	0.006277
17	F	Unclassified EE	6 months	CDKL5 FOXG1	GRIN1 / NM_000832.6	c.2504C>A / p.Ala835Asp	Not reported	*De novo*	0.933 / 0	3.97	Not present

F: female, M: male, EOEE: early-onset epileptic encephalopathy, EE: epileptic encephalopaty, NLES: Non-lesional epileptic spasms, SMEI: severe myoclonic epilepsy of infancy, dbSNP: single nucleotide polymorphism database, MAF: minor allele frequency, NA: not available, IVF: in vitro fertilisation, PolyPhen2: polymorphism phenotyping version 2, SIFT: sorting intolerant from tolerant, GERP: genomic evolutionary rate profiling, ExAC: exome aggregation consortium.

Informed parental consent for genetic testing was obtained in all cases. DNA samples were extracted from peripheral blood lymphocytes using standard procedures. The study was approved by the local ethics committee (Hospital Universitario Fundación Jiménez Díaz).

### Epilepsy panel

We designed 2 panels using Agilent’s SureDesign tool (www.agilent.com/genomics/suredesign) including genes that were known to be involved in epilepsy as a phenotypic feature according to the Online Mendelian Inheritance in Man (OMIM) database (http://www.ncbi.nlm.nih.gov/omim). These genetic panels cover exonic regions as well as exon–intron boundaries of the selected genes.

We designed a first panel comprising 83 genes (S1 Table) to screen a cohort of 44 patients. A second panel was designed with 106 genes (S2 Table) to screen 43 more patients. This second panel included new genes that have been more recently associated with epilepsy, excluding some genes contained in the first panel.

Theoretically, panel 1 included 17612 amplicons covering 873721 Mbp (99.42% of the region of interest), and panel 2 included 19597 amplicons, 1105 Mbp and 99.78%. Certain regions did not achieve a satisfying coverage and needed resequencing by Sanger.

### Target enrichment method

We used customized in-solution target enrichment followed by NGS to screen for variants in our 2 cohorts of patients. A library of all coding exons and intron-exon boundaries was prepared using a HaloPlex target enrichment kit (Agilent, Santa Clara, USA) following the manufacturer’s instructions. Briefly, we fragmented the human genome (the samples were digested by 16 different restriction enzymes to create a library of gDNA restriction fragments) and enriched for the coding regions of genes by using complementary highly specific biotinylated probes. HaloPlex probes are designed to hybridise selectively to fragments originating from target regions of the genome. and to direct circularisation of the targeted DNA fragments. Hybridized probes were captured with magnetic beads and target fragments were ligated to create circular DNA molecules. Subsequently, libraries were amplified by PCR, introducing unique index sequences that allow all pools to be sequenced together. Sequencing was performed using the NGS MiSeq Illumina sequencer (Illumina, Inc.). As an acceptance threshold value we selected a Q-score of 30, corresponding to a 1:1000 error rate.

### Bioinformatics tools

Fastq files from the sequencer were redirected to a custom pipeline for HaloPlex^™^ Target Enrichment System on the DNA nexus platform and/or to Agilent Surecall software.

Briefly, reads were aligned to the human reference genome (GRCh37/hg19) (http://hgdownload.cse.ucsc.edu/) with Burrows-Wheeler Aligner (BWA) [28] and variants were called using at least 2 of the 3 following variant callers: Genome Analysis Toolkit (GATK) [29–31], Freebayes [32] (both within the DNA nexus platform), and Base Alignment Quality (BAQ) Single Nucleotide Polymorphism (SNP) caller (within SureCall tool).

Variants passing quality filters were annotated separately against NCBI RefGene (http://www.ncbi.nlm.nih.gov) and ENSEMBL Variant Effect Predictor ver.72 (http://www.ensembl.org/info/docs/tools/vep).

### Prioritization of candidate genes

Variants were further filtered out to exclude all variants classified as synonymous, non-pathogenic, or with a frequency above 0.01 in control populations (data from dbSNP database (http://www.ncbi.nlm.nih.gov/projects/SNP/), 1000 Genomes Project (http://1000genomes.org), Exome Sequencing Project (http://evs.gs.washington.edu/EVS/), and Exome Aggregation Consortium (ExAC) (http://exac.broadinstitute.org/)). We attempted to estimate the putative pathogenic effect of non-reported suspected variants with conventional and freely available online tools, such as Polymorphism Phenotyping version 2 (Polyphen2) (http://genetics.bwh.harvard.edu/pph2/), Sorting Intolerant From Tolerant (SIFT) (http://sift.bii.a-star.edu.sg/), and HSF (http://www.umd.be/HSF/) [33–35]. PolyPhen2 scores of less than 0.15 are predicted to be benign, scores from 0.15 to 0.85 as possibly damaging, and scores greater than 0.85 are interpreted as probably damaging. SIFT scores of less than 0.05 are predicted to be deleterious and those greater than or equal to 0.05 are predicted to be tolerated. Also, we used Exomiser (http://www.sanger.ac.uk/resources/databases/exomiser/) [36], an online tool that functionally annotates and prioritises mutated genes using variant frequency, predicted pathogenicity, inheritance pattern, and model organism phenotype data as criteria. Scores are based on Mutation Taster [35], SIFT [37], Polyphen2 [34], and GERP [38]. GERP scores ranged from -12.3 to 6.17, with 6.17 being the most highly conserved.

Finally, the selected variants were evaluated within the context of their individual phenotype and clinical data. Putatively causative mutations were validated by conventional Sanger sequencing and tested by segregation analysis when possible.

### Criteria for pathogenicity

We classified a novel variant as pathogenic according to the international guidelines of the American College of Medical Genetics (ACMG) Laboratory Practice Committee Working Group [39].

## Results

We recruited, studied, and classified 87 patients with epilepsy mostly with an onset in the first year of life (68/87, 78.2%), and with developmental delay. Clinical diagnoses are summarized in Table 2. A large proportion of patients were unable to be classified, mainly due to incomplete clinical data (56/87). The patients were analyzed using a targeted next-generation custom gene panel. A mean coverage of 263× was obtained per sample (minimum 83× and maximum 443×), with 88% of bases covered at more than 30×. The percentage of read mapped to the reference genome was between 84.8% and 91.8%, with a mean of 88.3%.

**Table 2:** Clinical diagnosis in 87 patients with epilepsy and developmental delay.

Clinical diagnoses		*n*	%
Non-lesional epileptic spasms (NLES)		12	13.8%
Early-onset epileptic encephalopathy (EOEE)^a^		9	10.3%
Lennox-Gastaut syndrome (LGS)		6	6.9%
Landau-Kleffner syndrome (LKS)		1	1.1%
Severe myoclonic epilepsy in infancy (SMEI)		1	1.1%
Myoclonic-astatic epilepsy (MAE)		1	1.1%
Malignant migrating partial seizures of infancy (MMPSI)		1	1.1%
Unclassified epileptic syndromes	Epileptic encephalopathies (EEs)	44	50.6%
	Generalized epilepsies	8	9.2%
	Focal epilepsies	4	4.6%

^a^Early-onset epileptic encephalopathy includes Ohtahara syndrome (OS) and early myoclonic encephalopathy (EME).

After a stringent filtering procedure was carried out, a total of 18 presumed disease-causing variants in 12 genes were detected, including *KCNQ2* (n = 4), *CDKL5* (n = 3), *SCN1A* (n = 1), *PCDH19* (n = 1), *STXBP1* (n = 1), *SLC2A1* (n = 1), *ARX* (n = 1), *ALG13* (n = 1), *SYNGAP1* (n = 1), *GRIN1* (n = 1), *CHD2* (n = 1), and *POLG* (n = 2). We identified 3 (16.7%) frame-shift insertion-deletion, 2 (11.1%) putative splice site, and 13 (72.2%) missense variants, of which 12 (66.7%) arose *de novo* and 6 (33.3%) were novel.

Genomic evolutionary rate profiling (GERP) score showed that these variants affected highly conserved amino acids in mammals and were reported to be deleterious in the prediction programs used. In total, we were able to identify 18 disease-causing variants in 17 patients.

Of the 17/87 (19.5%) patients with positive findings, 10/44 (22.7%) had unclassified EEs, 5/9 (55.6%) had EOEE, 1/1 (100%) was diagnosed with severe myoclonic epilepsy of infancy (SMEI), and 1/12 (8.3%) was included in NLES group.

An overview of all detected variants is shown in Table 1.

It is worth noting that the positive cases in our panel had previously undergone different clinical and genetic tests, including karyotyping (41.2%, (7/17)), magnetic resonance imaging (MRI) (100%, (17/17)), metabolic screening (70.6%, (12/17)), mitochondrial DNA screening (23.5% (4/17)), comparative genomic hybridisation (CGH) array test (5.9% (1/17)), and sequential single-gene analysis (1–6 genes, see Table 1) (58.8% (10/17)).

Four point variants in *KCNQ2* were identified in 3 patients with EOEE and one patient with unclassified EE: c.602G>A (patients 1 and 2), c.601C>T (patient 3), and c.803T>C (patient 4). Two variants arose *de novo* (see Table 1). Parental DNA samples (either parent) were not available for patient 3, or no paternal DNA sample was available for patient 1 (both were born following *in vitro* fertilisation (IVF)).

Interestingly, a variant affecting the same codon as in patients 1 and 2 has been reported, though with a different base substitution (Arg>Cys) [40].

The variants c.601C>T and c.602G>A are located in the transmembrane S4 domain, and the c.803T>C variant is located in the pore-forming H5 domain of the protein. All 3 variants are predicted to be pathogenic.

We also detected a previously reported *STXBP1* heterozygous variant (c.1216C>T, rs796053367) in a patient diagnosed as having EOEE. This causative variant is located at exon 14, leading to the substitution of a conserved residue, R406, in domain 3b of *STXBP1*, and was not found in her parents [41, 42]. All patients described above had onset in the first 3 weeks of life and the electroencephalogram (EEG) showed a burst-supression pattern.

Three *de novo CDKL5* variants (c.52_53insT, c.377G>A, and rs267606715) presumed to be disease-causing were identified in 3 patients belonging to the unclassified EE group (see Table 1). The variants c.52_53insT and c.377G>A are located in the catalytic domain of the protein. Furthermore, the c.52_53insT variant affects the ATP-binding site and produces a truncated protein [43].

We have also identified pathogenic variants in *ALG13*, *GRIN1*, *ARX*, *SCN1A*, *PCDH19*, *SLC2A1*, *CHD2*, *SYNGAP1*, and *POLG* (see Table 1).

The variant found in *ALG13* (c.320A>G) in a patient with NLES who progressed to Lennox-Gastaut syndrome (LGS) has been previously reported (rs398122394) as pathogenic and is located in the region where glysosyltransferase activity resides.

Two causative *de novo* variants in *GRIN1* (c.2504C>A, p.Ala835Asp) and *ARX* (c.196G>A, p.Gly66Ser, rs1057518564) were found in a patient with an unclassified EE (patient 17) and in another diagnosed as EOEE (patient 15), respectively. The *GRIN1* variant affects the calmodulin-binding domain, a highly conserved domain of the N-methyl-D-aspartate (NMDA)-receptor 1 [44]. Variants in this domain disturb interactions with intracellular proteins, which may impair receptor function [45].

We identified a *de novo SCN1A* splicing variant (c.602+1G>A, rs794726827) in a patient with unclassified EE. This pathogenic variant affects the splice-donor site in intron 4 and is located in the S3 transmembrane segment of domain I of the SCN1A protein. Human Splicing Finder (HSF) does not predict a cryptic splice-site activation; therefore, this variant may lead to skipping of exon 4, resulting in an affected channel. We could not confirm the predicted consequences because RNA samples were not available.

A paternally inherited *PCDH19* variant (c.698A>G, p.Asp233Gly) was detected in one female diagnosed with SMEI. This novel variant is located in the first exon, which codifies the extracellular domain of the protein. The patient did not present any autistic features and her father was asymptomatic, contrasting with data reported by other authors [46, 47].

Patient 13 showed a heterozygous splice-site pathogenic variant (c.115-2A>G) in *SLC2A1*, which was confirmed as *de novo*. This variant affects the splice-acceptor site of the third exon but, according to the HSF, a cryptic splice site is activated. It causes a variation in the length of the exon, eliminating 9 nucleotides, which results in a loss of 3 amino acids in the protein. We could not test the functional consequence of this splice-site variant because the RNA samples were not available.

A G>A transition in the nucleotide 2317 in *CHD2*, which produces a Glu>Lys substitution in position 337, was found in patient 12, who had an unclassified EE. This change is located in the first chromodomain of the protein, likely affecting the remodeling of chromatin.

A novel disease-causing variant in *SYNGAP1* (c.333_334insG, p.Lys114GlufsTer38) was found in a patient diagnosed as unclassified EE. This variant was not found in the parents. The variant p.Lys114GlufsTer38 is located in the pleckstrin homology domain in the N-terminal segment of the protein, and generates a truncated protein.

Finally, we identified 2 reported pathogenic variants in *POLG* (c.156_158dupGCA, p.Gln52dup; c.2492A>G, p.Tyr831Cys) in a patient included as an unclassified EE. The first variant was inherited from his mother, though the heritability of the second mutation could not be confirmed. The patient presented a late-onset unclassified EE with posterior electrical and neuroimaging abnormalities compatible with those previously described in patients harboring variants in *POLG*.

## Discussion

NGS panels are now used widely in the clinical setting to identify genetic causes of epilepsy, replacing the traditional gene-by-gene approach. The genetic heterogeneity and the phenotypic overlap in severe epilepsies beginning in infancy and early childhood make multigene panel analysis a useful diagnostic tool.

Results from recent large studies incorporating NGS of patients with EE reveal that up to 30% of cases can be conclusively resolved with current technologies [48].

In this study, we describe the development of a Haloplex-based NGS assay in 87 patients with epilepsy and developmental delay. Applying this gene panel analysis, we were able to identify deleterious variants in 19.5% patients (17 of 87). Our results are in accordance with those previously reported by other authors, with diagnostic yields ranging between 10% and 48.5% [8, 23, 26, 27, 49–55]. Recent data show that *de novo* variants play an important role in EEs [56–58]. In our study, 12 out of 18 (66.7%) pathogenic variants were shown to be *de novo*.

We identified positive findings in most known prominent epilepsy genes such as *KCNQ2* [59], *CDKL5* [60], *STXBP1* [61], *SCN1A* [62, 63], *PCDH19* [64], *POLG* [65], *SLC2A1* [66], and *ARX* [67] and in others more recently associated with EE such as *ALG13* [56], *CHD2* [68], *SYNGAP1* [69], and *GRIN1* [70].

All of these genes are well established for severe pediatric epilepsies. We found a causative variant in 10 of 44 patients diagnosed with unclassified EE, the majority (8 of 10) with seizure onset in the first years of life, and 5 of 9 classified as EOEE. The overall positive rates were 14.3% and 55.6% in these groups of patients, respectively [53, 71, 72].

As mentioned above, the positive findings were related to genes well established as being causative of severe epilepsies beginning in infancy and early childhood and are consistent with the phenotypes of our patients, although the genotype could have been unsuspected.

In our panel analysis, we were unable to detect causative variants in 70 out of the 87 patients belonging to the NLES group, LGS, Landau-Kleffner syndrome (LKS), myoclonic-astatic epilepsy (MAE), and malignant migrating partial seizures of infancy (MMPSI) groups categorized as EEs, and in groups consisting of unclassified generalized and focal epilepsies. There are several reasons for these negative results. First, the patients were recruited for research purposes. Subsequent clinical and genetic studies identified the etiology in 12 cases: 9 patients showed structural brain abnormalities on MRI scan, 2 individuals carried a mitochondrial pathogenic variant, and 1 patient harboured a heterozygoous deletion in *PCDH19* that was not detected in the panel. The finding of a lesion, previously not detected, is not infrequent in the pediatric population mostly because of the difficulty to identify focal cortical dysplasia in late infancy and early childhood but also due to the increasing use of higher field MRI. Second, as we did not have detailed phenotypic data for 20 patients and many of these patients were studied at early stages of the disease, the final diagnosis may have been modified (in fact, during follow-up 1 patient was finally diagnosed with Jeavons syndrome). Some of these patients were recruited a substantial amount of time ago and it is likely that other clinical or genetic tests could shed light on the underlying etiology (e.g. a CGH-array in children with epilepsy associated to ID with/without dysmorphic features). Finally, the absence of any presumed disease-causing variant in 37 patients with intensive follow-up and without relevant clinical changes was probably due to the fact that the causative gene was not present in our design. On the other hand, it should be noted that the findings in the negative cases included in epileptic disorders with a low diagnostic yield are in accordance with data reported by other authors [8, 17, 23, 25, 49–54].

Our study confirms the last published findings reported by other authors [26, 53] regarding the diagnostic yield of genetic testing in patients with severe pediatric epilepsies (especially in patients with early-onset epileptic encephalopathies). Additionally, considering the high proportion of patients with unclassified epilepsies in our series, the results support the use of a multigene epilepsy panel for a hypothesis-free diagnostic approach. Despite the fact that the clinical presentations of the epileptic disorders frequently overlap and even when phenotypic data are scarce, this type of approach, which includes the most relevant epilepsy-associated genes, offers rapid testing with a good diagnostic yield.

In conclusion, our HaloPlex design demonstrates the utility of this gene panel approach to identify the cause of cases with some type of genetic epilepsy in infancy. The early identification of the underlying causative genetic alteration using NGS approach will provide prognostic information, influence therapeutic decisions and lead to the design of new drugs targeted to gene-specific defects [9, 10, 73, 74].

## Supporting information

S1 Table Genes in the first panel of epilepsy. (DOC)

S2 Table Genes in the second panel of epilepsy. (DOC)
