# Supplementary material for: Molecular diagnosis of patients with epilepsy and developmental delay using a customized panel of epilepsy genes
Source: PLoS One. 2017 Nov 30;12(11):e0188978. doi: 10.1371/journal.pone.0188978 (PMC5708701; doi:10.1371/journal.pone.0188978)
Supplement: S1 Table — (DOC) [file pone.0188978.s001.doc]

**S1 Table.** **Genes in the first panel of epilepsy.**

| **Gene** | **Interval** | **Gene** | **Interval** |
| --- | --- | --- | --- |
| ALDH7A1 | chr5:125880557-125930990 | KCNQ2 | chr20:62037897-62103916 |
| ARHGEF9 | chrX:62854747-63005513 | KCNQ3 | chr8:133141409-133492879 |
| ARX | chrX:25022687-25033954 | KCTD7 | chr7:66093952-66105481 |
| ATP1A2 | chr1:160085552-160111212 | LGI1 | chr10:95517802-95557660 |
| BRD2 | chr6:32940576-32948595 | MAGI2 | chr7:77648532-79082736 |
| CACNA1A | chr19:13318027-13617138 | MAPK10 | chr4:86938281-87115654 |
| CACNA1B | chr9:140772286-141016551 | MBD5 | chr2:149216228-149270610 |
| CACNA1E | chr1:181452781-181768070 | ME2 | chr18:48422091-48473654 |
| CACNA1H | chr16:1203638-1271094 | MECP2 | chrX:153295718-153363222 |
| CACNB4 | chr2:152695533-152955625 | MFSD8 | chr4:128841685-128886388 |
| CDKL5 | chrX:18525117-18671764 | NEU1 | chr6:31827396-31830653 |
| CLCN2 | chr3:184064294-184079367 | NHLRC1 | chr6:18121550-18122937 |
| CLN3 | chr16:28488737-28503180 | NTNG1 | chr1:107691116-108023562 |
| CLN5 | chr13:77565987-77575204 | PCDH19 | chrX:99551175-99663695 |
| CLN6 | chr15:68500378-68522022 | PLCB1 | chr20:8113199-8862596 |
| CLN8 | chr8:1703844-1734838 | PNKP | chr19:50364405-50370561 |
| COL4A1 | chr13:110802610-110959474 | PNPO | chr17:46018942-46024248 |
| CSTB | chr21:45193983-45196250 | POLG | chr15:89859882-89877085 |
| CTSD | chr11:1774633-1785189 | PPP1R3C | chr10:93389584-93392826 |
| CHRNA2 | chr8:27319046-27328675 | PPT1 | chr1:40539633-40563010 |
| CHRNA4 | chr20:61977990-61992617 | PRICKLE1 | chr12:42853511-42866418 |
| CHRNB2 | chr1:154540421-154548508 | PRICKLE2 | chr3:64084627-64184703 |
| DCX | chrX:110544815-110654302 | PRRT2 | chr16:29824276-29826059 |
| EFHC1 | chr6:52285109-52357239 | SCARB2 | chr4:77082766-77134796 |
| EPM2A | chr6:145822619-146057260 | SCN1A | chr2:166847655-166930231 |
| FOXG1 | chr14:29236386-29238055 | SCN1B | chr19:35521625-35530705 |
| FOXP1 | chr3:71008298-71247632 | SCN2A | chr2:166152234-166246434 |
| GABRA1 | chr5:161277717-161324528 | SCN2B | chr11:118037502-118047246 |
| GABRB3 | chr15:26792840-27018971 | SCN5A | chr3:38591712-38674898 |
| GABRD | chr1:1950763-1961821 | SCN8A | chr12:52056502-52201313 |
| GABRG2 | chr5:161494906-161580474 | SCN9A | chr2:167055082-167168366 |
| GBA | chr1:155204686-155211003 | SLC25A22 | chr11:791815-795106 |
| GLB1 | chr3:33038437-33138677 | SLC2A1 | chr1:43392612-43424422 |
| GPR56 | chr16:57684100-57697594 | SMS | chrX:21958843-22012569 |
| GPR98 | chr5:89854613-90459817 | SPTAN1 | chr9:131328920-131395713 |
| GRIN2A | chr16:9856906-10274368 | SRPX2 | chrX:99901220-99926084 |
| GRIN2B | chr12:13715617-14019242 | STXBP1 | chr9:130374583-130453236 |
| HEXA | chr15:72636318-72668413 | SYN1 | chrX:47432163-47479227 |
| HEXB | chr5:73980986-74017100 | TPP1 | chr11:6635677-6640731 |
| KCNA1 | chr12:5020445-5022132 | TSC1 | chr9:135771522-135804359 |
| KCNJ10 | chr1:160011083-160012422 | TSC2 | chr16:2098517-2138711 |
| KCNMA1 | chr10:78646924-79397500 |  |  |
