# Supplementary material for: Molecular diagnosis of patients with epilepsy and developmental delay using a customized panel of epilepsy genes
Source: PLoS One. 2017 Nov 30;12(11):e0188978. doi: 10.1371/journal.pone.0188978 (PMC5708701; doi:10.1371/journal.pone.0188978)
Supplement: S2 Table — (DOC) [file pone.0188978.s002.doc]

**S2 Table. Genes in the second panel of epilepsy.**

| **Gene** | **Interval** | **Gene** | **Interval** |
| --- | --- | --- | --- |
| ALDH5A1 | chr6:24495187-24533950 | KCNH6 | chr17:61600685-61623273 |
| ALDH7A1 | chr5:125880647-125931092 | KCNQ1 | chr11:2466211-2870350 |
| ALG13 | chrX:110924336-111003237 | KCNQ2 | chr20:62037987-62104003 |
| ALG3 | chr3:183960292-183967323 | KCNQ3 | chr8:133141499-133493014 |
| ANO4 | chr12:101188364-101520858 | KCNT1 | chr9:138594021-138684017 |
| ARFGEF2 | chr20:47538265-47649746 | LGI1 | chr10:95517556-95557570 |
| ARHGEF17 | chr11:73019653-73078835 | MAGI2 | chr7:77648622-79082900 |
| ARHGEF9 | chrX:62857898-63005436 | MBD5 | chr2:148778570-149270520 |
| ARX | chrX:25022777-25034075 | MECP2 | chrX:153295808-153363198 |
| ATP1A2 | chr1:160085510-160111122 | NBEA | chr13:35516414-36245138 |
| CACNA1A | chr19:13318117-13617284 | NEDD4L | chr18:55711600-56063511 |
| CACNA1H | chr16:1203231-1271004 | NEU1 | chr6:31827486-31830719 |
| CACNA2D2 | chr3:50402087-50540902 | NHLRC1 | chr6:18121640-18122847 |
| CASK | chrX:41379663-41782297 | NTNG1 | chr1:107682619-108023472 |
| CBL | chr11:119076976-119170501 | P2RX2 | chr12:133195356-133198568 |
| CDKL5 | chrX:18443715-18671674 | PCDH19 | chrX:99551265-99665281 |
| CHD2 | chr15:93443541-93567945 | PIGA | chrX:15339618-15353686 |
| CHRNA2 | chr8:27319136-27336823 | PIGN | chr18:59713079-59854299 |
| CHRNA4 | chr20:61978080-61992758 | PIGO | chr9:35089079-35096608 |
| CHRNB2 | chr1:154540247-154548418 | PIGQ | chr16:619958-633644 |
| CNTN2 | chr1:205012330-205042903 | PLCB1 | chr20:8112902-8862506 |
| CSNK1G1 | chr15:64464120-64648452 | PNKP | chr19:50364495-50370832 |
| CSTB | chr21:45194073-45196266 | PNPO | chr17:46018879-46024158 |
| DCX | chrX:110544905-110655470 | POLG | chr15:89859972-89878036 |
| DEPDC5 | chr22:32149927-32302493 | PRRT2 | chr16:29823399-29827212 |
| DNM1 | chr9:130965653-131017003 | RBFOX1 | chr16:6069122-7760757 |
| EFHC1 | chr6:52284984-52360593 | RBFOX3 | chr17:77086955-77512240 |
| ELP4 | chr11:31531287-31805082 | RYR3 | chr15:33603167-34157437 |
| EPM2A | chr6:145946821-146057001 | SCN10A | chr3:38738830-38835511 |
| FASN | chr17:80037009-80056116 | SCN1A | chr2:166847745-167005652 |
| FLNA | chrX:153577207-153603016 | SCN1B | chr19:35521582-35530615 |
| FOXG1 | chr14:29236268-29239493 | SCN2A | chr2:166095902-166246344 |
| FOXP1 | chr3:71008388-71633150 | SCN2B | chr11:118037592-118047347 |
| FOXP2 | chr7:113726355-114333837 | SCN5A | chr3:38591802-38691174 |
| GABBR2 | chr9:101052856-101471489 | SCN8A | chr12:51985010-52201223 |
| GABRA1 | chr5:161274187-161324438 | SCN9A | chr2:167055172-167232507 |
| GABRB3 | chr15:26792930-27018945 | SHANK3 | chr22:51113060-51169750 |
| GABRD | chr1:1950758-1961731 | SLC25A12 | chr2:172641774-172750826 |
| GABRG2 | chr5:161494638-161580384 | SLC25A22 | chr11:791905-798279 |
| GNAO1 | chr16:56225241-56388975 | SLC2A1 | chr1:43392702-43424857 |
| GPR56 | chr16:57653900-57697504 | SLC35A2 | chrX:48760705-48769245 |
| GRIN1 | chr9:140033599-140062304 | SPTAN1 | chr9:131314827-131395623 |
| GRIN2A | chr16:9856996-10276621 | SRPX2 | chrX:99899153-99925994 |
| GRIN2B | chr12:13715707-14133032 | ST3GAL3 | chr1:44173194-44396847 |
| HCN2 | chr19:589883-616484 | STX1B | chr16:31004132-31021839 |
| HEXA | chr15:72636408-72668530 | STXBP1 | chr9:130374476-130453146 |
| HEXB | chr5:73980959-74017010 | SYN1 | chrX:47432253-47479266 |
| HNRNPU | chr1:245017742-245027837 | SYNGAP1 | chr6:33387837-33419693 |
| IQSEC2 | chrX:53263391-53350532 | SZT2 | chr1:43855546-43916161 |
| KCNA1 | chr12:5019063-5027432 | TBC1D24 | chr16:2525137-2550969 |
| KCNA2 | chr1:111136505-111174106 | TPP1 | chr11:6635767-6640702 |
| KCND2 | chr7:119913712-120387922 | ULK1 | chr12:132379269-132405916 |
| KCNH2 | chr7:150642443-150675412 | ULK2 | chr17:19679652-19771249 |
